# Water Pipes

**Published:** 1866-04

**Authors:** 


### WATER PIPES.

It is becoming more common every year to supply farm-houses with water through lead pipes, distributed through the building. Some waters, as the Schuylkill, which supplies Philadelphia, contain an element which forms on the inside of the pipe, a film which is absolutely impervious by the water, and protects the lead against all corrosion or chemical change. And in cities and large towns, where the water is kept running almost incessantly, time is not allowed for chemical action on the lead; where the same water, through the same pipes, would produce speedy sickness in a farm-house. It is water stagnant in a lead pipe which causes mischief; so that every faucet should be allowed to run the water waste for at least one minute the first thing in the morning, especially in the kitchen. Still, comparatively little harm would result under ordinary circumstances if while the leaden pipes are laid, the most special care should be taken as to these points :

Allow no angles in the pipe.

Let every piece of pipe which is horizontal lie perfectly straight.

Have all curves as large as possible.

Have no indentation on the outside of the pipe, for this may cause a projection on the inside.

Be at great pains that no pebble or other thing shall be left in a pipe at the time of its being laid.

All these look to one point, that is, the prevention of any sediment lodging at any one point, for where this occurs, there will be found the elements of corrosion and chemical change, from which the poisoning comes.

